# Analyzing the Effect of Social Distancing Policies on Traffic at Sinchon Station, South Korea, during the COVID-19 Pandemic in 2020 and 2021

**DOI:** 10.3390/ijerph19148535

**Published:** 2022-07-13

**Authors:** Nam-gun Kim, Hyeri Jang, Seungkeun Noh, Ju-hee Hong, Jongsoon Jung, Jinho Shin, Yongseung Shin, Jongseong Kim

**Affiliations:** 1 Seoul Metropolitan Government Research Institute of Public Health and Environment, Seoul 13818, Korea; chunil2@seoul.go.kr (N.-g.K.); hrjang@seoul.go.kr (H.J.); bluethy@seoul.go.kr (S.N.); ghdghd@seoul.go.kr (J.-h.H.); sjlab@seoul.go.kr (J.J.); sjh81@seoul.go.kr (J.S.); shiny64@seoul.go.kr (Y.S.); 2 Department of Biomedical Sciences, College of Medicine, Korea University, Seoul 02841, Korea

**Keywords:** social distancing policies, change traffic volume, COVID-19 pandemic

## Abstract

The COVID-19 pandemic is recognized as one of the most serious global health problems, and many countries implemented lockdown measures to mitigate the effects of the crisis caused by this respiratory infectious disease. In this study, we investigated the relationship between social distancing policies and changes in traffic volume in Sinchon Station, South Korea. We used an official COVID-19 report provided by the Korea Disease Control and Prevention Agency (KCDA) and Seoul Metropolitan Government (SMG) to review social distancing policies, and the changes in traffic patterns before and during the COVID-19 pandemic between January 2020 and November 2021 were analyzed. Our study reveals that the changes in the overall traffic patterns from acceleration phases to deceleration phases of COVID-19 were related to the alert levels of social distancing policies implemented to tackle the situation resulting from the COVID-19 pandemic. Herein, we found that a significant decline in traffic volume took place from August to September 2020 (13.5–19.7%, weekday; 19.4–31.7%, weekend), from December 2020 to January 2021 (20.0%−26.6%, weekday; 26.8–34.0%, weekend), and from July to September 2021 (3.2–13.1%, weekday; 38.3–44.7%, weekend) when compared to the corresponding periods in 2019 (paired *t*-test; *p* < 0.001). The results of this study provide strong support for the effectiveness of Seoul’s preemptive measures, namely, the central government’s intensive social distancing campaign, in managing and reducing the impact of the pandemic situation based on the precise analysis of 10 types of facilities.

## 1. Introduction

Cases of COVID-19, a disease resulting from infection by a novel positive-strand RNA coronavirus (SARS-CoV-2), were first reported in Wuhan City, Hubei province, China, in December 2019. The rapid spread of cases around the world led to COVID-19 being declared a global pandemic by the World Health Organization (WHO) in March 2020 [1,2]. Because of the emergence of new coronavirus variants in many places and their strong infectivity, the COVID-19 pandemic is recognized as one of the most serious global health problems [3,4]. Therefore, most countries implemented lockdown measures, applying various restrictions such as closing their borders, reducing business activity, delaying school opening times, and restricting traffic [5]. As a result, drastic changes in socioeconomic conditions were widely observed [6].

Many studies have reported South Korea’s prevention strategy for controlling the spread of COVID-19 as being successful [7,8,9]. According to previous reports, there are various reasons contributing to the country’s success. After the country’s response to the outbreak of Middle East respiratory syndrome (MERS), which was the second largest outbreak after being first identified in Saudi Arabia in 2015, the Korea government prepared 48 reforms to boost the infection prevention system [10,11]. The effective use of smart technology, such as advanced mobile technologies and various apps, facilitated the subsequent development of intensive contact tracing and the management of contacts, real-time information sharing, and appropriate treatments for patients [12,13]. Other relevant studies stated that the country’s success is the result of its rich human resources and infrastructure in addition to constructive relationships between government authorities and large numbers of test cases [11,14].

Authors located in many different areas around the world have analyzed large-scale mobility data sets, such as using big data approaches, to identify the routes of COVID-19 transmission and to evaluate the effectiveness of health authorities’ policies for disease control during the COVID-19 pandemic [5,15,16]. One study examining the influencing factors of COVID-19 in Wuhan in Hubei, China, showed that there are clear relationships between population flow and the COVID-19 transmission rate [17]. Another study focusing on the association between mobility changes and the transmission of COVID-19 using country-level data in the USA concluded that social distancing has a considerable influence on the reduction in COVID-19 cases [16]. According to Yabe et al., there is a strong connection between the reduction in social contacts and noncompulsory measures in Tokyo, Japan [18]. These authors indicated that social distancing policies are important for alleviating COVID-19 infection.

**Figure 1:**
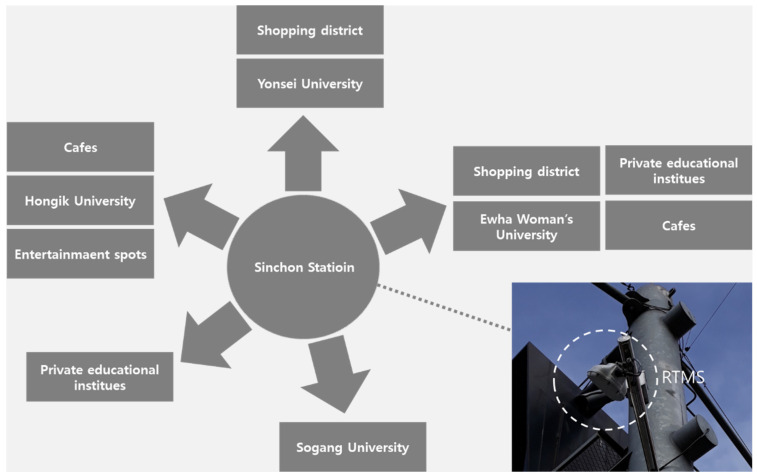
A simplied illustration of 5-way Sinchon Intersection (research site).

This study aimed to analyze the social distancing policies implemented by the central government and the Seoul Metropolitan Government’s measures in responding to the respiratory infectious disease crisis and their relationship with changes in traffic volumes in a selected area. Sinchon Station (South Korea), which is on Line 2 of the Seoul Metropolitan Subway, was selected as our research site. A previous study which analyzed the association between commuting flow and pandemic mitigation policies hints at the importance of the correlation, resulting in the reduction (10–20%) of commuting flow in South Korea [19]. Another study also reported that the reduction in economic activity and electricity usage was related with the decrease in human activity [20,21]. As shown in Figure 1, this area was considered adequate for observing the effects of social distancing policies—i.e., nonpharmaceutical interventions, including refraining from attending social gatherings, staying at home, school closures, and cancellation of public events—based on two main factors, namely that (1) universities, such as Yonsei University, Ehwa Woman’s University, Sogang University, and Hongik University, are concentrated in this area, and (2) this site is a popular shopping district that hosts street performances, cafes, private educational institutes, and entertainment spots [22].

## 2. Methods

### 2.1. Study Design

This quasi-experimental study consists of a review of social distancing policies from central and local governments and an analysis of traffic data before and during the COVID-19 pandemic at Sinchon Station between January 2020 and November 2021. Traffic volumes in January 2020 and November 2021 were compared with those for the corresponding period in 2019 to identify the impacts of lockdown on traffic volume change. This method was somewhat based on the previous approach that evaluated the association between traffic volume and COVID-19 prevalence and further examined the reduction of traffic accident by lockdown [23,24,25].

### 2.2. Data Collection

The data on traffic volume were collected from a real-time microwave sensor (RTMS) Sx-300 (Figure 1). This system is a radar-based device that measures vehicles per lane and speed in real time. Traffic count data from RTMS were analyzed by analytics software (S4605M, S4605A (Seoul, Korea)). The difference in the distribution of matched traffic data was tested using a paired *t*-test (IBM SPSS Statistics 24 (Armonk, NY, USA)) [26]. Social distancing policies in South Korea were analyzed using the official COVID-19 report provided Ministry of Health and Welfare (https://www.mohw.go.kr/eng/nw/nw0101ls.jsp?PAR_MENU_ID=1007&MENU_ID=100701 (accessed on 25 May 2022)) and Seoul Metropolitan Government (SMG) (https://english.seoul.go.kr/category/policy-information/welfare-health-security/welfare-health-security-news/ (accessed on 25 May 2022)). COVID-19 patient data in Seoul city were collected from the statistics from the SMG on a daily basis.

## 3. Results

### 3.1. Seoul-Style Precision Disease Control and Prevention for 10 Types of Facilities

Due to the sharp increase in the number of confirmed COVID-19 cases in Seoul, the SMG implemented “Seoul-style precision disease control and prevention for 10 types of facilities” measures on 8 December 2020. With the implementation of this policy regarding facilities that are vulnerable to COVID-19 transmission, the volume of traffic was observed to decrease at a fast rate. Thus, we reviewed studies examining measures similar to the preventive measures implemented by the SMG that had a significant effect on reducing traffic volume; these studies are detailed in Table 1.

#### 3.1.1. Religious Facilities

Numerous COVID-19 transmission cases have been linked to religious facilities, such as sites of worship, as well as religious gatherings in Malaysia, Italy, Iran, Singapore, and South Korea [27]. In particular, in Daegu, South Korea, a religious group meeting was a key factor in the rapid propagation that led to over 5000 cases in February and March 2020 [28]. Since religious sites may facilitate the rapid propagation of SARS-CoV-2 in both direct (between an individual with the virus and a susceptible individual) and indirect ways (between a susceptible individual and an object carrying the virus), the temporary closure of sites of worship can help in the prevention of rapid transmission [27,29].

#### 3.1.2. Workplace

Employees in the workplace are vulnerable to COVID-19 virus infection due to the high number of people. Person-to-person transmission of the COVID-19 virus occurs more easily through people with fever and respiratory symptoms than through those who are asymptomatic. Hence, employers prepare prevention strategies for high-risk employees based on scientific guidelines [30].

#### 3.1.3. Bath Facilities

SARS-CoV-2 in the form of aerosol particles survives longer than other respiratory viruses, and it can remain infectious for up to 3 h. Bath facilities, such as spa pools, are among the main reasons for severe epidemics of respiratory viruses due to the transmission of aerosols generated at such facilities [31,43].

#### 3.1.4. Indoor Sports Facilities

An interpersonal distance of 1.5 m between individuals may be an effective measure against COVID-19 when people are standing and are all wearing face masks. However, despite the effectiveness of social distancing, it is necessary to consider the potential aerodynamic effects caused by the physical movement, such as when people walk and run. An aerodynamics study suggests that the distance between an exposed and an infected person together with their traveling speeds is a key factor determining whether substantial droplet exposure occurs [32,33].

#### 3.1.5. Restaurants and Cafes

A recent analysis of aerosol transport in a restaurant using computational fluid dynamics showed a correlation between regions with a high aerosol exposure index and infection patterns. Local recirculation flow caused by thermal plumes and air conditioner ventilation flow was observed to increase airborne infection risk [34].

#### 3.1.6. Door-to-Door Sales

The occurrence of an outbreak between 27 June and 16 July 2020 was related to door-to-door retailers in Gwangju metropolitan city (South Korea), and it seemed that a small gathering of members triggered the first surge in COVID-19 cases. This outbreak indicated that most of those confirmed positive for COVID-19 were not following appropriate disease control and prevention measures, such as mask wearing at all times and maintaining a 2 m distance with others [35].

#### 3.1.7. Karaoke Rooms

On February 2020, in Guangzhou (China), it was confirmed that an outbreak of COVID-19 occurred at a karaoke party organized by a WeChat group. A study was conducted, and the authors deduced that this outbreak resulted from individuals singing in close contact with each other and sharing a microphone. Thus, the authors recommended that, during the spread of this novel coronavirus disease, the government should temporarily shut down such facilities and the owners should improve ventilation performances and ensure mask-wearing behavior in karaoke rooms [36].

#### 3.1.8. Internet Cafes

Cases of COVID-19 related to internet cafes involved a middle school student in Busan (South Korea) in February 2020 and men (age range: 1–79 years) in Seoul (South Korea) in March 2020. Customers remain in internet cafes for long periods of time to play games and eat food without wearing face masks, thereby generating droplets in poorly ventilated areas and allowing viruses to easily spread at these facilities. Therefore, it is essential that preventive measures, such as social distancing among customers, disinfection procedures for subsequent customers, and swift contact identification, are carried out at such facilities [37,38].

#### 3.1.9. Private Academies

According to Kim, Eun Young, et al., among 127 pediatric COVID-19 cases in South Korea, 14% were related to cram schools and private lessons [39]. Since children and adolescents appear to be more susceptible to SARS-CoV-2 infection, though with milder or no symptoms in cases of a positive result than in adult cases, they may be a significant factor for the community spread of COVID-19 [40,44]. Thus, it is important to understand the epidemiological characteristics related to children and adolescents and to implement appropriate prevention strategies, such as social distancing, mask wearing, and ensuring adequate ventilation in indoor environments [44].

#### 3.1.10. Social Welfare Facilities

Cases of COVID-19 related to nosocomial transmission involved 66 inpatients (15%) in London in March and April 2020 and patients at several hospitals in Tokyo (Japan) in May 2020 [41,42]. In hospital-acquired cases, the possible routes of infection are direct contact with patients residing in the same bay and indirect contact via facility sharing or staff movement, and a high rate of mortality has been observed [41,42]. As such, the authors highlighted the necessity of precautionary measures to prevent transmission from various routes in hospital, such as diagnostic tests for all patients upon admission and the surveillance of staff and patients [41].

### 3.2. Traffic Decrease and Lockdown

As shown in Table 2, we analyzed the mean monthly decrease in the rate of traffic volume alongside lockdown measures implemented by the Korean government and the SMG at Sinchon Station. The decrease in traffic was analyzed as a percentage between 1 February 2020 and 31 November 2021, and we compared this with the trend in the corresponding period in 2019. The difference in the traffic distribution decrease before and after lockdown (2019 versus 2020; 2019 versus 2021) was statistically significant (reduction of traffic volume was compared on a monthly basis (weekdays or weekends) and the difference was tested using paired *t*-test statistics; *p* < 0.001). The lockdown measure included in this study is the social distancing policy that was implemented to prevent the large-scale community spread of COVID-19 in the public and private sectors.

During the occurrence of large-scale local community transmission related to religious groups, the health authorities raised the public alert level, which comprises four tiers, to the highest level of “serious” on 23 February 2020, and social distancing was first introduced for COVID-19 on 29 February 2020 [14,45,46]. In the following month (March), although Seoul had a relatively small number of new positive cases, the SMG preemptively launched a campaign with “Hold up! Let’s Take a Break from Social Life” as its slogan [47]. The Korean government announced strengthened social distancing rules to avoid closing various facilities, such as religious facilities, indoor sports halls, and entertainment venues. During this period, traffic on weekdays and weekends decreased by 10.3 and 3.9%, respectively, in February and by 17.5 and 17.1%, respectively, in March.

By 6 May 2020, the Korean government implemented “transition from social distancing to distancing in daily life” in an attempt to partially ease social distancing measures while still preventing the further spread of the disease as an effort to return to everyday life. This measure consisted of permitting activity at low-risk places, such as recreation parks and closed-door sporting events, and forbidding activity at high-risk facilities, such as religious facilities and fitness centers and gyms [46,48]. During May, traffic on weekdays and weekends decreased by 12.9 and 9.2%, respectively.

On 16 August 2020, due to the easing of social distancing rules, the number of newly confirmed COVID-19 patients steadily increased at the community level, e.g., at religious facilities, hospitals, and daycare centers; thus, the social distancing guide level was raised to Level 2 in Seoul city and Gyeonggi province [47,49]. In line with central government measures, the SMG designated the period from 30 August to 6 September as the “10 Million Citizens Stop Week” to enhance the existing Level 2 measures. These measures were as follows: take-out and delivery from franchise coffee shops were only permitted between 9:00 p.m. and 5:00 a.m.; offline classes at private academies were prohibited; convalescent individuals were required to self-isolate. As a result of these measures, traffic on weekdays and weekends decreased by 13.5 and 19.4%, respectively, in August and by 19.7 and 31.7%, respectively, in September.

In October 2020, as the number of new confirmed cases dropped, the central government implemented a policy that downgraded the social distancing measures to Level 1, effective as of 12 October [50]. However, the Seoul Metropolitan Area maintained some Level 2 measures for high-risk facilities. At that time, traffic on weekdays and weekends decreased by 5.8 and 16.6%, respectively.

On 19 November 2020, with the increasing trend in the local transmission of COVID-19, the social distancing measures were elevated to Level 1.5 in the Seoul Metropolitan Area [50]. Nevertheless, on 24 November 2020, (https://www.seoulsolution.kr/en/content/8859 (accessed on 25 May 2022)) due to pressure from the rapid spread of COVID-19 cases, the central government raised the social distancing measures to Level 2 for the Seoul Metropolitan Area. Moreover, SMG declared an “Emergency pause period for 10 million citizens” and implemented the “Seoul-style precision disease control and prevention for 10 types of facilities”, which are facilities with a high risk of infection. Furthermore, in the following month (December), the central government raised the social distancing measures to Level 2.5 for the Seoul Metropolitan Area. As a result of stronger measures, traffic on weekdays and weekends dropped by 11.3 and 14.0%, respectively, in November, and by 20.0 and 26.8%, respectively, in December.

Due to the high possibility of spreading the disease through gatherings and travel to other regions of the country during the Lunar New Year holiday, Level 2.5 social distancing measures in the capital region were maintained until 14 February 2021 [51]. After that, on 15 February, the disease control authorities announced the plan to ease social distancing regulations to Level 2 in response to reduced COVID-19 activity and social fatigue. During this period, traffic on weekdays and weekends decreased by 15.0 and 30.2%, respectively.

The Korean government announced the plan to support the return to everyday life for those who received a COVID-19 vaccine by easing social distancing regulations, including implementing exemptions from some of the disease prevention rules in three stages in June, July, and then October based on the vaccination situation. However, this plan was postponed due to the rising rates of infection [52]. During this period (June 2021), traffic on weekdays and weekends decreased by 10.5 and 26.7%, respectively.

On 12 July 2021, in response to the widespread outbreaks of COVID-19 in various locations, the social distancing measures were elevated to the highest Level of 4 in the Seoul Metropolitan Area. At this level, private gatherings of a maximum of two people were only permitted after 6 p.m., and all events, excluding weddings and funerals, were officially prohibited (http://english.seoul.go.kr/level-4-new-social-distancing-guideline/ (accessed on 25 May 2022)). During the implementation of strict measures in July, traffic volumes on weekdays and weekends decreased by 13.1 and 38.8%, respectively.

The central government introduced the “Living with COVID-19” plan, which began on 1 November 2021 (https://english.seoul.go.kr/covid-19-steady-return-to-pre-pandemic-life-starting-nov-1/ (accessed on 25 May 2022)). Its purpose was to gradually phase out the social distancing measures in three phases at 6-week intervals. Furthermore, the health authorities implemented a vaccine pass policy to allow visitors to enter multiuse facilities, such as gyms, singing rooms, and clubs. Visitors had to show either proof of vaccination or a negative result certificate (RT-PCR) to gain access [52]. In November, the last month of observation, traffic on weekdays and weekends decreased by 20 and 26%, respectively.

**Figure 2:**
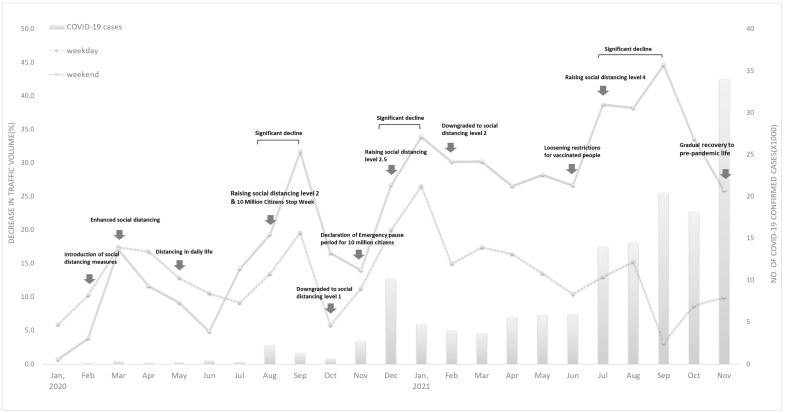
Monthly number of confirmed COVID-19 cases in Seoul (source: https://www.seoul.go.kr/coronaV/coronaStatus.do (accessed on 25 May 2022)) and monthly decrease rates of traffic volume at Sinchon Station. The left y-axis corresponds to rate of traffic volume decrease, and the right y-axis corresponds to the number of confirmed COVID-19 cases. The black line and gray dotted line indicate the rates of traffic volume decrease between 2019 and 2020, and between 2019 and 2021, respectively.

Figure 2 shows an analysis of the time-series data, which shows the decrease in traffic volume as a percentage and observed COVID-19 case trends during the research period of 2020–2021 when compared to the equivalent period from 2019. The Figure shows both weekday and weekend data separately. In general, the decrease in traffic volume accompanies the strengthening of social distancing measures, and there is an overall increase in the number of new COVID-19 cases, which grew rapidly up to September 2021. In particular, the reduction in traffic volume was more pronounced on weekends. After that, the trends in traffic volume from September to November on weekends and weekdays tended to diverge. Weekdays shifted to an increasing trend, while weekends showed a decreasing trend.

## 4. Discussion

During the COVID-19 outbreak, analyzing the changes in roadway traffic volume via a traffic measuring system has played an important role in identifying the impacts of social distancing policies on traffic change patterns, which is a reflection of changes in people’s mobility. This study demonstrates that the overall traffic change patterns from acceleration phases to deceleration phases of COVID-19 were related to alert levels of social distancing policies implemented to tackle the pandemic situation, and it suggested that various prevention and control policies carried out by the SMG and central government contributed to mitigating COVID-19 transmission in Seoul.

A significant decline in traffic volume took place from August to September 2020 (13.5–19.7%, weekday; 19.4–31.7%, weekend), from December 2020 to January 2021 (20.0–26.6%, weekday; 26.8–34.0%, weekend), and from July to September 2021 (3.2–13.1%, weekday; 38.3–44.7%, weekend) when compared to the corresponding periods in 2019. At that time, the traffic volume decreased on both weekdays and weekends, but the reduction rate was more distinct on weekends. This could mainly be attributed to changing patterns of activities. This finding is consistent with a study conducted by Yang, Shujuan, et al. (2020), which reported that COVID-19 lockdowns lead to a decrease in leisure time activity [53].

The main factors influencing these decreasing trends in traffic volume are as follows: (1) the Seoul-style precision disease control and prevention for 10 types of facilities measures (from December 2020 to January 2021) and (2) raising the level of social distancing measures (from July to September 2021). Our findings indicate that the response to the COVID-19 pandemic by local and central governments effectively prevented the transmission of infection. This change corresponds to the phenomena reported by Parr, Scott, et al. (2020), who analyzed highway volume patterns before and during the COVID-19 crisis in Florida [54]. Another relevant study conducted by Brodeur et al. (2020) investigated the effect of safer-at-home policies and found that these policies led to a decline in traffic collisions in several US states [55].

First, precise measures for 10 types of facilities based on the incidence of COVID-19 cases were implemented in Seoul, and the results show both that traffic volume first sharply decreased in December 2020 and that COVID-19 cases showed a steep decline in January 2021. This result could be explained by the fact of the timely response to COVID-19 in Seoul, whereby the appropriate action—as determined from the precise analysis of vulnerable factors and infection prevalence statistics from 2514 facilities from 12 August to 20 November 2020—was carried out. According to these statistics (https://www.seoulsolution.kr/en/content/8855 (accessed on 25 May 2022)), religious facilities had the highest number of cases and were the most susceptible to infectious diseases, followed by the workplace, nursing homes and hospitals, indoor sports facilities, and restaurants and cafes. These results are consistent with the previous report by Effiong, Fortune, et al. (2021), who analyzed gatherings (at schools, religious centers, and markets) and their relationship with COVID-19 transmission [56]. Therefore, it appears that aerosol and droplets linked to a facility’s spatial characteristics and activity have played a key role in the spread of the new coronavirus.

Second, due to the rapid increase in the number of confirmed cases, the highest level of social distancing measures was implemented in the Seoul Metropolitan Area on 12 July 2021. As a result of the implementation of this policy, the highest reduction in traffic was observed on weekends, while a relatively small decrease in traffic was observed on weekdays, and unlike the previous pattern, the number of confirmed cases showed a tendency to increase from August to November 2021. The changes in this pattern could be explained by the substitution of public transportation with private vehicles for daily commutes [57,58] and increased COVID-19 vaccination rates, which can lead to declining participation in social distancing as a result of beliefs concerning returning to normal pre-COVID-19 life [59].

The shift to a decreasing trend of confirmed COVID-19 cases in Seoul occurred during the following periods: (1) from March to April 2020, (2) from August to October 2020, and (3) from December 2020 to March 2021. These reductions happened on the tandem with local and central government’ policies to address community transmission (Table 2). Our findings illustrate that working partnerships between the central and local governments in fighting the pandemic crisis seem to exert a synergistic outcome. Also, it could be an effective model for future outbreak of contagious disease without enforcing a strict lockdown related to civil liberties. According to previous studies, the South Korean government should act as a good mediator of multiple sectors of society’s response to COVID-19 outbreak and central and local collaboration is an important element for the successful control of epidemic [12,60,61].

The one caveat of the present study is the lack of vehicle classification in analyses. The research only included overall traffic volumes without verifying the types of vehicles passing at specific points during the study period. Since different types of vehicles can disproportionately impact traffic volume during lockdown, further research will need to analyze vehicle categories, such as motorcycles, passenger cars, buses, and single-unit or multi-unit trucks, to determine the statistical significance of the traffic percentage change.

## 5. Conclusions

In conclusion, this study analyzed traffic change patterns at Sinchon Station, South Korea, during the COVID-19 pandemic after various prevention and control policies implemented by the SMG and central government. Traffic volumes for 2020 and 2021 significantly declined in three major periods (① from August to September 2020, ② from December 2020 to January 2021, and ③ from July to September 2021) when lockdown measures were imposed, as in the case of Sinchon Station. Based on the precise analysis of 10 types of facilities, Seoul’s preemptive measures and the central government’s intensive social distancing campaign, which were carried out to mitigate the impact of the pandemic situation, were found to contribute to the effective management of infectious disease by decreasing traffic volumes. Also, a close collaboration between local and central government for the control of infections led to the drop in confirmed COVID-19 cases. Thus, our results hint at the importance of government policy to prevent or at least attenuate social crises such as COVID-19 pandemic.

## Figures and Tables

**Table 1:** Implementation of Seoul-style precision disease control and prevention for 10 types of facilities and related studies.

Category	Description	Relevant Studies
Religious facilities	- Participation in regular church and temple services at 20% capacity - Recommendation of switching participation in regular church and temple services at 20% capacity to remote worship	- Yezli, Saber et al., 2021 [27] - Chang, Min Cheol et al., 2020 [28] - Vermeer, Paul et al., 2020 [29]
Workplace	- Less than 50% working capacity and remote working recommended - Recommendation to take preemptive testing for COVID-19 when people suffer similar symptoms 2 or more times a day or when 2–3 or more patients are confirmed	- Rafeemanesh et al., 2020 [30]
Bath facilities	- Prohibition of eating food	- van Doremalen et al., 2020 [31] - Yezli, Saber et al., 2021 [27]
Indoor sports facilities	- Suspension of operation after 9 p.m. and operation of shower rooms - Distance of 2 m maintained	- Blocken, Bert, et al., 2020 [32] - Setti, Leonardo et al., 2020 [33]
Restaurants and cafes	- Distance of 2 m between users maintained when waiting for orders and no talking while eating - Installation of partition on counters or a distance of at least 1 m maintained between counters and customers	- Liu, Han, et al., 2020 [34]
Door-to-door sales	- No serving of refreshments such as coffee and snacks - All meetings ending within 20 min	- Kang, Seung-Ji, 2021 [35]
Karaoke rooms	- Maximum of 1 user per 4 m^2^ per room - Mandatory measuring of user’s body temperature	- Gu, Yuzhou, et al., 2021 [36]
Internet cafes	- High partition to block droplets - Prohibition of eating and drinking	- Kang, Yun Jung, et al., 2020 [37] - Cho Ryok Kang, et al., 2020 [38]
Private academies	- Less than 50% user capacity	- Kim, Eun Young, et al., 2020 [39] - Dong, Yuanyuan, et al., 2020 [40]
Social welfare facilities	- Restrictions on visiting, going out, and staying out overnight	- Rickman, Hannah M., et al., 2021 [41] - Tagashira, Yasuaki, et al., 2021 [42]

**Table 2:** Traffic volume decrease per month between February 2020 and November 2021 at Sinchon Station.

Periods		Decrease Rate (%) ^1)^		Social-Distancing-Related Policies
		Weekday	Weekend	
2020	Feb.	10.3	3.9	- Raise crisis alert level to highest (23 February 2020) ^3)^ - Introduction of social distancing measures (29 February 2020) ^3)^
	Mar.	17.5	17.1	- Public campaign under the slogan “Hold up! Let’s Take a Break from Social Life” (2 March 2020) ^2)^ - Implementation of enhanced social distancing (22 March 2020) ^3)^
	May	12.9	9.2	- The transition from “social distancing” to “distancing in daily life” - (6 May 2020) ^3)^
	Aug. ^4)^ Sep. ^4)^	13.5 19.7	19.4 31.7	- Elevation of social distancing measures to level 2 (16 August 2020) ^3)^ - Implementation of 10 Million Citizens Stop Week (30 August 2020) ^2)^
	Oct.	5.8	16.6	- Social distancing measures downgraded to level 1 (30 October 2020) ^3)^
	Nov.	11.3	14.0	- Elevation of social distancing in Seoul Metropolitan Area to Level 1.5 - (19 November 2020) ^3)^ - Declaration of emergency pause period for 10 million citizens - (24 November 2020) ^2)^
	Dec. ^4)^ Jan. 2021 ^4)^	20.0 26.6	26.8 34.0	- Elevation of social distancing measures in Seoul Metropolitan Area to level 2.5 - (8 December 2020) ^3)^
2021	Feb.	15.0	30.2	- Social distancing measures downgraded to level 2 in Seoul Metropolitan Area (15 February 2021) ^3)^
	Jun.	10.5	26.7	- Some incentives that ease restrictions for vaccinated people provided (1 July 2021) ^3)^
	Jul. ^4)^ Aug. ^4)^ Sep. ^4)^	13.1 45.3 3.2	38.8 38.3 44.7	- Elevation of social distancing in Seoul Metropolitan Area to level 4 - (12 July 2021) ^3)^
	Nov.	10.0	26.0	- Gradual recovery to pre-pandemic life through the easing of social distancing measures (1 November 2021) ^3)^

^1)^ Decrease rate (%) = (average traffic per month (2020 or 2021) ÷ (average traffic per month (2019) × 100). ^2)^ These measures were implemented by the Seoul Metropolitan Government. ^3)^ These measures were implemented by the Korean government. ^4)^ Significant decline in traffic volume.

## Data Availability

Data used in this research are available on the website Seoul Metropolitan Government at ” https://www.seoul.go.kr/coronaV/coronaStatus.do (accessed on 25 May 2022) and Ministry of Health and Welfare at “https://www.mohw.go.kr/eng/nw/nw0101ls.jsp?PAR_MENU_ID=1007&MENU_ID=100701” (accessed on 25 May 2022).
